# Genome assembly, characterization, and mining of biosynthetic gene clusters (BGCs) from *Chlorogloeopsis* sp. ULAP02 isolated from Mt. Ulap, Itogon, Benguet, Philippines

**DOI:** 10.3389/fgene.2024.1422274

**Published:** 2024-08-30

**Authors:** Libertine Rose S. Sanchez, Danica Pearl M. Untiveros, Maria Theresa T. Tengco, Ernelea P. Cao

**Affiliations:** Plant Molecular Biology and Genetics Laboratory, Institute of Biology, College of Science, University of the Philippines Diliman, Quezon City, Philippines

**Keywords:** Chlorogloeopsis sp., cyanobacteria, genome mining, biosynthetic gene clusters (BGCs), antimicrobial properties

## Introduction

Cyanobacteria are known for their wide distribution, even in extreme environmental conditions, primarily due to their ability to produce secondary metabolites or bioactive compounds specialized for survival. These autotrophic Gram-negative bacteria have been studied for the discovery of novel antimicrobial compounds such as terpenes, alkaloids, phenols, carbohydrates, polyketides, and peptides (Micallef et al., 2015b; Srivastava et al., 2022).

Recent advancements in genome sequencing have enabled screening of genes responsible for encoding a novel natural compound with biological significance through genome mining. This was applied previously in some species from subsection V cyanobacteria where a broad range of impressive bioactive compounds were synthesized by giant non-ribosomal peptide synthetases (NRPS), smaller ribosomally-synthesized and post-translationally modified peptides (RiPPs), polyketides synthases (PKS), terpenes, alkaloids, fatty acids and UV-absorbing compounds (Micallef et al., 2015a; 2015b). These cyclopeptides can be of ribosomal or non-ribosomal origin. Among all, the cyclic lipopolypeptides (CLPs) have the reported widest range of bioactivities (Saurav et al., 2022). They possess a peptidic backbone, a protein or non-protein amino acids attached to a fatty acid tail that forms a ring structure (Götze and Stallforth, 2020), which promote integration into the membrane of target microbes (Fiedler and Heerklotz, 2015).

Most of the cyanobacterial BGCs are non-ribosomal peptides (NRPs), polyketides (PKs), or hybrid peptide-polyketide. NRPs in cyanobacteria are synthesized by multidomain mega-enzymes NRPS assembled outside of ribosomal translation. Their complex and diverse molecular scaffolds contribute to their biotherapeutic potentials as antibacterial, antiviral, antiprotozoal and antitumor agents. On the other hand, the RiPPs superfamily has rapidly increased its genomic sequence data of natural products from the linear chains of precursor peptides, consisting of leader peptides and core peptides produced by ribosomes (Do and Link, 2023; Ongpipattanakul et al., 2022). RiPPs have very high antimicrobial potential since they target bacterial cell wall especially those of Gram-negative bacteria that eventually leads to cell death (Cao et al., 2021). Polyketides are synthesized in a Claissen-type acyl condensation pathway by the NRPS or PKS as a substrate (Entfellner et al., 2022; Fischbach and Walsh, 2006).

Furthermore, the hybrid NRPS-PKS are also prolific cyanobacterial products. Some of these BGCs have been identified from multiple genomes across numerous genera and species. Among these include microcystin from *Microcystis*, *Anabaena*, *Fischerella* and *Planktothrix*; nodularin from *Nodularia*; cylindrospermopsin from *Cylindrospermopsis* and *Oscillatoria*; curacin and barbamide from *Lyngbya* and *Moorea*; anabaenopeptilides from *Anabaena* sp.; anabaenopeptins from *Microcystis*, *Anabaena*, *Planktothrix* and *Nodularia* (Micallef et al., 2015b). Lanthipeptides are RiPPs abundantly found in cyanobacteria that exhibit high antimicrobial potential attributed to its thioether amino acids lanthionine and methyllanthionine (Fu et al., 2023; Tang and van der Donk, 2012)

*Chlorogloeopsis* is a member of Nostocales with varying morphology reportedly influenced by the culture condition. For example, the *Chlorogloeopsis* sp. ULAP02 analyzed in this study is similar to the morphology described for culture grown under photoautotrophic conditions with cells dividing up to three planes (Evans et al., 1976). Meanwhile, limited genomic data and studies on bioactive metabolites are available for this cyanobacterium. To date, there are only three uploaded whole genome sequences of Chlorogloeopsis sp. at the National Center for Biotechnology Information (NCBI) isolated from soil, non-acidic hot spring and thermal spring. The strain used in the present study was isolated from copper mine tailings. This study aims to construct and characterize the whole genome sequence of *Chlorogloeopsis* sp. ULAP02 isolated from a small-scale private mining site in Itogon, Benguet, Philippines. Various genome mining tools were employed to identify BGCs with associated antimicrobial properties. These results can add to the existing genomic database information about another freshwater cyanobacterium which we previously isolated from a copper mine tailing environment. Furthermore, genome mining leads to more productive cultivation techniques and a faster discovery approach of secondary metabolites in general.

## Value of data

This study presents an assembled genome of a unicellular *Chlorogloeopsis* sp. ULAP02 isolated from a stressful condition in a mining environment. Results of the phylogenetic analysis showed a cluster separate from the common *Chlorogloeopsis fritschii,* which might indicate a different species or a novel species. Furthermore, this paper lists BGCs with known antimicrobial properties mined from the generated genome. Results of this study may serve as reference for research on the organism’s genome and provides significant insights on the synthesis and potential biotherapeutic applications of these bioactive compounds.

## Methods

### Isolation, culture maintenance, and morphological characterization

*Chlorogloeopsis* sp. ULAP02 was isolated from tailings samples collected from a small-scale, private copper mining site at Mt. Ulap in Ampucao, Itogon, Benguet, Philippines (16.2505°N, 120.6775°E). Environmental samples obtained were processed in the Plant Molecular Biology and Genetics Laboratory (PMBGL) of the Institute of Biology, College of Science, University of the Philippines, Diliman, Quezon City. The isolation was performed using classical microbiological techniques in both solid and liquid BG-11 media amended with Kanamycin and Nystatin at a concentration of 10 μg/mL and 100 μg/mL, respectively. All cultures were maintained in the same laboratory and exposed to white fluorescent lamps for a 12-h light-dark cycle.

The morphology of *Chlorogloeopsis* sp. ULAP02 isolates was examined using a light compound microscope (Labomed LB-221, United States). The observed morphological characters such as cell size, shape, presence of sheath and color were compared to the descriptions from the literature (Anagnostidis and Komarek, 2005). The photomicrographs were taken with the aid of ScopeImage 9.0.

### Genomic DNA extraction, initial identification, and whole genome sequencing

Genomic DNA of the unialgal culture was extracted using the Zymo Research *Quick* DNA Fungal/Bacterial Miniprep Kit (Irvine, CA, United States), following the manufacturer’s instructions. The quality (A260/280) and quantity of the extracted DNA were assessed using a spectrophotometer (Epoch™) and visualized on 1% agarose gel. Initial molecular identification was performed by amplifying and sequencing the 16S *rRNA* gene region following the protocol of Nübel, 1997). Upon confirmation, the genomic DNA was sent to Macrogen Inc., South Korea for whole genome shotgun sequencing (2 × 150 Paired-End) on the Illumina NovaSeq platform.

### Phylogenetic analysis

The 16s rRNA sequence of the isolate was aligned with available sequences of *Chlorogloeopsis* (*Chlorogloeopsis* sp.) (7) and *C. fritschii* (10)] and other closely-related cyanobacteria genera [*Anabaena* (2), *Calothrix* (1), *Cylindrospermopsis* (1), *Cylindrospermum* (1), *Desmonostoc* (2), *Fischerella* (1), *Halotia* (2), *Hapalosiphon* (1), *Nodularia* (1), *Nostoc* (2), *Scytonema* (1), *Tolypothrix* (1)] for phylogenetic analysis in MEGA X: Molecular Evolutionary Genetics Analysis (Kumar et al., 2018) using MUSCLE. Maximum likelihood (ML) tree was generated using Kimura-2-Parameter Model *(+G + I)*, as suggested by the model testing in the same software. Partial 16S rRNA sequence of *Gloeobacter violaceus* was used as the outgroup.

### Genome assembly, taxonomic classification, and functional analysis

The quality of the paired-end reads was assessed using FastQC v0.12.1 (Andrews, 2010) and preprocessed using BBDuk v38.22 (Bushnell, 2014) to remove adapters and trim low-quality reads. Additionally, PRINSEQ v0.20.4 (Schmieder and Edwards, 2011) was employed to filter out low-complexity reads.

*De novo* genome assembly was conducted using SPAdes v3.15.3 on the KBase web platform (https://kbase.us) (Nurk et al., 2017). The process utilized multiple k-mer sizes (21, 33, 55, 77, 99, and 127) to optimize the quality and accuracy of assembly. The assembled genome was evaluated for completeness and contamination using CheckM v1.0.18 (Parks et al., 2015), a genome quality assessment tool based on lineage-specific marker genes.

The Genome Taxonomy Database Tool Kit (GTDB-Tk) v1.7.0 (Chaumeil et al., 2022) was used for the taxonomic classification of the assembled cyanobacterial genome. Functional annotation was then performed using the RASTtk v1.073 (Overbeek et al., 2005; Brettin et al., 2015) annotation pipeline, which incorporates a variety of computational methods to predict coding sequences, assign functions, and annotate genomic features.

### Genome mining of biosynthetic gene clusters (BGCs)

The genome of *Chlorogloeopsis* sp. ULAP02 as well as the RefSeq genomes of the three *Chlorogloeopsis* species available in NCBI [GCF_015272425.1, GCF_000317265.1, GCF_000317285.1] were subjected to different genome mining tools to identify and explore BGCs comprehensively.

The webtools antiSMASH 6.0 (Blin et al., 2019) and PRISM4 v4.4.5 (Skinnider et al., 2017) were used to predict gene clusters associated with secondary metabolite synthesis. Meanwhile, ketosynthase (KS) domains and condensation (C) domains were identified using the online server NaPDoS2 (Klau et al., 2022). RiPPMiner (Agrawal et al., 2017), a machine learning webserver, was also used to determine Ribosomally synthesized Post translationally modified Peptides (RiPPs). Finally, DeepBGC v0.1.29 (Hannigan et al., 2019) was employed to detect BGCs that may encode novel biological functions. Default parameters were applied to all the tools unless otherwise stated.

## Results and discussion

### Morphology of *Chlorogloeopsis* sp. ULAP02

**FIGURE 1:**
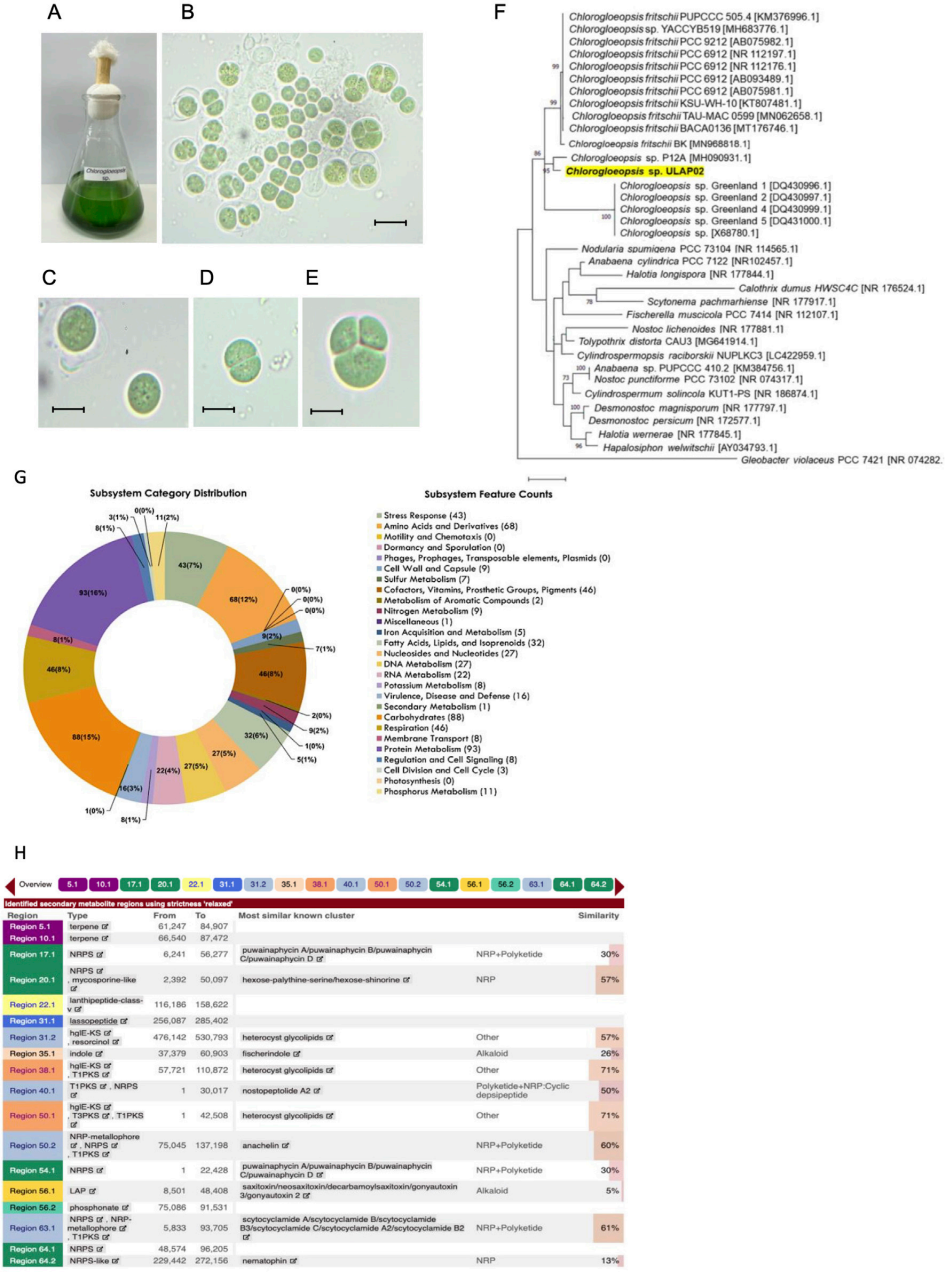
Morphological, molecular characterization and genome mining of biosynthetic gene clusters (BGCs) in *Chlorogloeopsis* sp. ULAP02. **(A)** Blue-green isolates in BG-11 medium. **(B)** Clusters of unicellular cells. **(C)** Single cell with a diameter of 17–21 μm. **(D)** Cell division in one plane at right angle producing two cells. **(E)** Cell division in irregular planes producing three cells of varying sizes. Microscopic figures were observed at 100X with a scale= 25 μm. **(F)** The Maximum Likelihood (ML) phylogenetic tree of *Chlorogloeopsis* sp. ULAP02 (highlighted in yellow) was generated using 16S rRNA gene sequencing based on Kimura-2-Parameter model (+G+I) generated by MEGA X. The local isolate *Chlorogloeopsis* sp. ULAP02 had a high bootstrap value of 86 that clustered with other *Chlorogloeopsis* sp. **(G)** The pie chart represents the cellular subsystem category and the number of protein-coding genes (in parentheses) that are predicted to be involved in the cellular process using the RASTtk. **(H)** Putative BGCs associated with antimicrobial metabolites, of which, heterocyst glycolipids, NRP+Polyketide and NRP are the most abundant.

The *Chlorogloeopsis* sp. grown in BG-11 medium appeared as deep blue-green cells that do not form mats (Figure 1A). Microscopic examination revealed a unicellular cyanobacterium that tends to form clusters (Figure 1B). Each cell is enclosed in a conspicuous colorless sheath with a diameter ranging from 17 to 21 μm (Figure 1C). Cells divide at right angles forming equal halves (Figure 1D) or irregularly in 1–3 planes (Figure 1E).

### Phylogenetic analysis

The partial 16S rRNA gene sequence of the local isolate *Chlorogloeopsis* sp. ULAP02 had a high percentage identity (96.12%) with the available *Chlorogloeopsis* sequences in NCBI. Furthermore, the local isolate clustered with other *Chlorogloeopsis* sp. with a high bootstrap value of 86 (Figure 1F), supporting the morphological identification. A separate cluster was formed by *C. fritschii.* Hence, the local isolate was not fully described up to the species level since it may belong to another species or a novel species yet to be identified.

### Genome assembly, taxonomic classification, and functional analysis

The whole genome sequencing using the Illumina NovaSeq platform generated 23,723,010 reads. After preprocessing, *de novo* assembly, and binning, 68 contigs were recovered. The cyanobacterial genome has an estimated size of 7,738,087 bp and a GC content of 40.90%, which is comparable to other *Chlorogloeopsis* genomes in NCBI as shown in Table 1.

**TABLE 1:** Summary of putative BGCs of *Chlorogloeopsis* ULAP02 using different genome mining tools.

Identification		*Chlorogloeopsis* sp.	*Chlorogloeopsis fritschii*.		
Strain		ULAP02	C42_A2020_084	PCC 9212	PCC 6912
NCBI RefSeq Assembly		GCF_038404745.1	GCF_015272425.1	GCF_000317265.1	GCF_000317285.1
Type of environment	Isolation source	Copper mine tailings	Non-acidic hot spring microbial mat	Water, thermal spring	Soil
	Geographical location	Philippines	Chile	Spain	India
Genome Features	Estimated genome size (Mb)	7.7	7.1	7.6	7.8
	GC Content (%)	41	41.5	41.5	41.5
	Number of contigs	68	241	188	161
	N50 (kb)	227.5	46.6	118	123.9
	L50	12	45	19	20
	Genes	6,774	6,354	6,853	6,951
	Protein coding genes	6,577	6,152	6,674	6,763
	Completeness (%)	99.64%	99.27%	99.70%	98.97%
	Contamination (%)	3.77%	1.72%	2.18%	2.18%
	Sequencing Platform	Illumina NovaSeq	Illumina HiSeq	454	454
	Genome coverage	100x	16.7x	25x	27x
	Assembly level	Scaffold	Contig	Contig	Contig
	Assembly method	SPAdes v. 3.15.3	SPAdes v. v3.13.0	GS De Novo Assembler v. 2.3	GS De Novo Assembler v. 2.0.01.14
Genome Mining Tools	**AntiSMASH**				
	Terpene	2	2	2	2
	NRPS	4	5	4	4
	NRPS-like	1	4	4	4
	Lanthipeptide class V	1	1	1	1
	Lasso peptide	1	1	-	-
	hgIE-KS	3	3	3	2
	Resorcinol	1	1	1	1
	Indole	1	1	1	1
	T1PKS	1	4	4	4
	T3PKS	1	1	1	1
	Linear azole(in)e-containing peptide (LAP)	1	—	—	—
	Phosphonate	—	1	1	1
	Lanthipeptide class II	—	—	2	2
	NRP-metallophore	—	1	1	1
	Mycosporine-like	—	1	1	1
	Trans-AT-PKS-like	—	—	1	1
	**deepBGC**				
	Polyketide	15	18	16	15
	Saccharide	9	18	18	13
	NRP	10	6	6	6
	RiPP	5	-	9	11
	Terpene	2	6	4	4
	Antibacterial	56	99	98	87
	Cytotoxic	4	5	9	7
	Inhibitor	1	—	1	1
	**PRISM**				
	NRP	7	5	5	5
	Prochlorosin	1	2	3	3
	Lassopeptide	1	1	—	-
	Class II Lantipeptide	—	—	2	2
	Resorcinol	1	—	1	1
	Polyketide	6	6	5	5
	Maleimide indolocarbazole	1	1	1	1
	Phosphonate	1	1	1	1
Genome Mining Tools (cont.)	RiPPMiner	Lassopeptide	Lassopeptide	Lanthipeptide B	Lanthipeptide B
	NaPDoS2				
	Ketosynthase (KS) Domain				
	Bacillus subtilis FAS	1	1	2	2
	Jamaicamide	2	3	2	2
	Photobacterium PUFA	2	3	3	3
	Shewanella PUFA	1	—	—	—
	Nosperin	2	—	1	1
	Puwainaphycin	1	1	1	1
	Macrolactin	1	—	—	—
	Schizochytrium PUFA	2	1	1	1
	Eschericoli FAS	1	-	—	—
	Moritella PUFA	1	2	2	2
	Cylindrocyclophane	—	1	1	1
	Disorazole	—	1	1	1
	Hectochlorin	—	1	1	1
	Nostopeptolide	—	2	—	-
	Salinomycin	—	1	1	1
	Swinholide	—	1	—	—
	Difficidin	—	—	1	1
	**Condensation (C) Domain**				
	Microcystin	11	4	2	2
	Nostopeptolide	13	8	7	7
	Tubulysin	2	—	—	—
	Viomycin	1	—	—	—
	Nodularin	—	1	1	1
	Pyoverdine	—	1	1	1
	Anabaenopeptilide	—	—	1	1

The results suggest that the isolate *Chlorogloeopsis* sp. ULAP02 belongs to the phylum Cyanobacteria based on taxonomic lineage obtained from the clade-specific marker gene sets. The completeness of the assembled genome was validated using CheckM tool giving a precise 99.76% completeness with only 1.04% contamination. Based on 68 genomes, 520 gene markers, and 415 marker sets, 510 single-copy genes were identified. There was a single orphan gene cluster and 9 heterogeneous genes. All 520 genes were grouped into a single bin.

The isolated strain *Chlorogloeopsis* sp. ULAP02 generated by GTDB-tk was predicted to belong to Phylum Cyanobacteriota, Class Cyanophyceae, Order Nostocales, Family Chlorogloeopsidaceae, and Genus *Chlorogloeop*sis.

RASTtk annotations were grouped according to their likelihood to be involved in a cellular process or subsystem (Figure 1G). Following the pie chart counterclockwise, subsystem categories are listed in the legend from top to bottom. Among all the cellular subsystem components, the (first) Protein Metabolism, (second) Carbohydrates, and (third) Amino Acids and Derivatives were the biggest groups, with 93, 88, and 68 annotated protein-coding genes, respectively.

### Prediction and putative identification of BGCs

Genome mining reveals high diversity of BGCs in *Chlorogloeopsis* sp. ULAP02, which is consistent with the *C. fritschii* found in the NCBI database. *Chlorogloeopsis* sp. ULAP02 was isolated from a copper mine tailing environment while the other *C. fritschii* were also isolated from freshwater sources except for strain PCC 6912, which was isolated in a soil habitat in India (Table 1). NRPs, PKS, RiPP lanthipeptides and lasso peptides are the major secondary metabolites of *Chlorogloepsis* sp. ULAP02 putatively identified in all mining tools namely: antiSMASH, DeepBGC, PRISM4, RiPPMiner and NaPDoS2, with the latter providing the greatest number of secondary metabolites. There are eight identified gene clusters common to antiSMASH, DeepBGC and PRISM4 namely: terpene, NRP, NRPS-like, resorcinol, TiPKS, T3PKS, phosphonate and lasso peptide. The latter is the only cluster predicted in RiPPMiner. Lasso peptide is a class of RiPPs with a unique lariat-like structure responsible for heat and chemical resistance, antimicrobial properties and resistance to protease degradation (Meyer et al., 2023; Zeng et al., 2022). In comparison with the *Chlorogloeopsis fristchii* found in the NCBI database (Table 1), lasso peptide was not detected in strains PCC9212 and PCC 6912 using the 454-sequencing platform. Whereas, trans-AT-PKS-like, Class II lantipeptide, difficidin, and anabaenopeptilide were not detected in an Illumina platform. Perhaps the more sophisticated Illumina Novaseq6000 compared to Illumina Hi-seq was able to sequence the linear azol (in)e- containing peptide, RiPP, resorcinol, Shewanella PUFA, nosperin, macrolactin, Eschericoli FAS, tubulysin and viamycin at 100x genome coverage.

Figure 1H summarizes the putatively identified secondary metabolites, type of BGCs, most similar known cluster, and the percent similarity to the region in the genome of *Chlorogloepsis* sp. ULAP02 using antiSMASH. A total of 18 BGCs were identified and characterized as terpene, NRPS, NRP-metallophore, mycosporine-like, Lanthipeptide Class V, lasso peptide, hgIE-KS, Resorcinol, Indole, T1PKS, T3PKS, LAP, and phosphonate (Supplementary Figures S1–S18). Most of the BGCs identified were assigned to hybrid NRPS-PKS, which have been found to be structurally and functionally diverse across cyanobacteria (Kudo et al., 2023). The hits of the database search were found to be BGC of puwainaphycin A, B, C and D; nostopeptolide A2, anachelin and scytoclamide A, A2, B, B2, B3 and C, with the latter having the highest 61% similarity to known clusters. Whereas the lowest percentage similarity to alkaloids is fischerindole and saxitoxin at 26% and 5%, respectively.

Scytoclamide is a hybrid PK-NRP isolated from *Scytonema hoffmanni* PCC 7110. It is a laxaphycin with a unique macrocyclic lipopeptides acting synergistically to show antiproliferative and antifungal activities. They are CLPs with macrocycles made up of either 11 amino acids (type A Laxaphycin) or 12 amino acids (type B laxaphycin) (Heinilä et al., 2020). Scytoclamide might have been present in *Chlorogloeopsis* sp. UPAL02 since it also possesses a hydrophobic alkaloid scytonemin that are usually observed among closely related species where *Scytonema* and *Chlorogloeopsis* belong to order Nostocales.

Puwainaphycins are widespread hybrid PK-NRP in cyanobacteria. They are CLPs consisting of a β-fatty amino acid and a 10-membered peptide ring. Puwainaphycin A, B, C and D were isolated from the soil bacterium *Cylindrospermum alatosporum* CCALA 988 (Mareš et al., 2014). They are homologous CLPs with high antifungal and cytotoxic activities (Saurav et al., 2022).

Another BGC mined with 60% similarity to the hybrid PK-NRP is anachelin from the first genuine siderophore of *Anabaena cylindrica* PCC 722 (Hoffmann et al., 2003). It also has a distinct structure being an alkaloid bound to a polyketide through a tripeptide of l-Thr, d-Ser and l-Ser. Anachelin H is the most common form consisting of a terminal salicylamide in its polyketide (Årstøl and Hohmann-Marriott, 2019). Being an iron-metal chelator, it has a diverse medical applications as antimalarial, antimicrobial, or anticancer drugs (Passari et al., 2023). The presence of anachelin siderophore in *Chlorogloeopsis* sp. ULAP02 might be due to its coping mechanism under iron limitation while it is living in a copper mine tailing.

Nostopeptolide A2, on the other hand, is a hybrid PK-NRP cyclic depsipeptide from a cryptophycin-producing terrestrial *Nostoc* sp. GSV22. Both nostopeptolides A1 and A2 possess a butyric group, and nine amino acid residues and an acetate-derived unit linked by ester and peptide bonds (Hoffmann et al., 2003). Its capacity to inhibit hepatoxin-induced apoptosis is a probable indication of its potential as potent antitoxin (Fidor et al., 2019). Nostopeptolide in *Chlorogloeopsis* sp. ULAP02 might be express in its free-living state and is able to repress hormogonia formation.

BGCs assigned to NRP include hexose-palythine-serine/hexose-shinorine at 57% and nematophin at 13% from *Heteroscytonema crispum* UCFS10 and *Xenorhabdus netophila* ATCC 19061, respectively.

Analysis in the NaPDoS2 predicted 14 CDSs related to metabolite production in ketosynthase (KS) domain pathways and 27 CDSs related to metabolite production in condensation (C) domain pathway.

The PRISM4 analysis identified three distinct gene clusters prochlorosin, maleimide and indolocarbazole. Prochlorosin is a lanthipeptide produced by an oligotrophic *Prochlorococcus*. They are unicellular organisms, similar to *Chlorogloeopsis* sp. ULAP02, that live in extremely diluted concentrations and were found to contain natural products as antimicrobial defenses (Tang and van der Donk, 2012). Indolocarbazole metabolite was extracted from *Nostoc sphaericum* EX-5-1 that showed an antiviral activity against type 2 Herpes-simplex virus (Sánchez et al., 2006).

## Conclusion

This study sequenced and assembled the genome of the freshwater *Chlorogloeopsis* sp. ULAP02 isolated from a copper mine tailing site. The genome size of 7,738,087 bp and GC content of 40.90% are comparable to other *Chlorogloeopsis* genomes in NCBI. This is the fourth genome assembly for the family Chlorogloeopsidaceae, where the two assembled data of *C. fritschii* were isolated from Germany and another *C. fritschii* from Chile. This is the first assembled genome of a *Chlorogloeopsis* strain isolated from the tropical region that could serve as a potential source of biosynthetic gene clusters for scytonamide, puwainaphycins, nostopeptolide and prochlorosin among others that possess a wide array of antimicrobial properties. Further experimentation and product extraction is suggested for the actual characterization and chemical structure of the putative *in silico* substances revealed through genome mining.

## Data Availability

The original contributions presented in the study are publicly available. This data can be found here: https://www.ncbi.nlm.nih.gov/genbank/, accession number SAMN38792795.
