# Supplementary material for: Genome assembly, characterization, and mining of biosynthetic gene clusters (BGCs) from Chlorogloeopsis sp. ULAP02 isolated from Mt. Ulap, Itogon, Benguet, Philippines
Source: Front Genet. 2024 Aug 30;15:1422274. doi: 10.3389/fgene.2024.1422274 (PMC11392904; doi:10.3389/fgene.2024.1422274)
Supplement: Supplementary file 1 [file Image1.pdf]

Supplementary information for:

Genome assembly, characterization and mining of BGCs with potential antimicrobial properties from *Chlorogloeopsis* sp. ULAP02 isolated from Mt. Ulap, Itogon, Benguet, Philippines

Supplementary Figures:

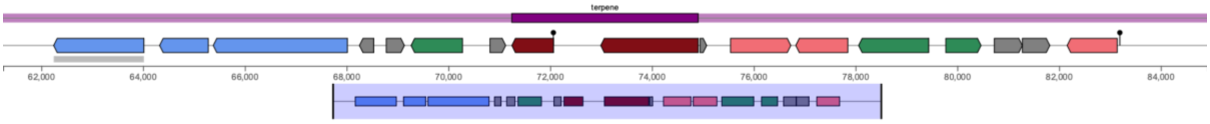

Figure S1. Location: 61,247 – 84,907 nt. (total: 23,661 nt); terpene

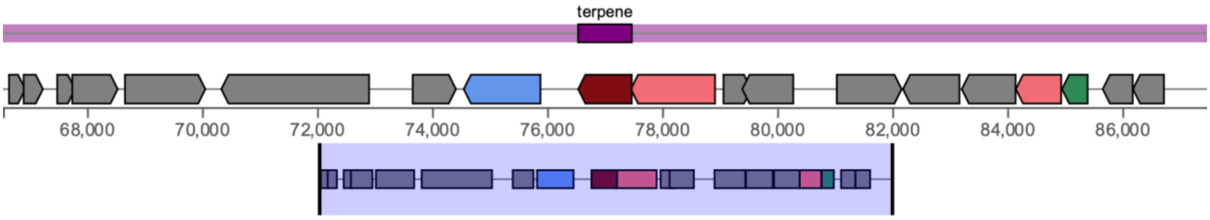

Figure S2. Location: 66,540 – 87,472 nt. (total: 20,933 nt); terpene

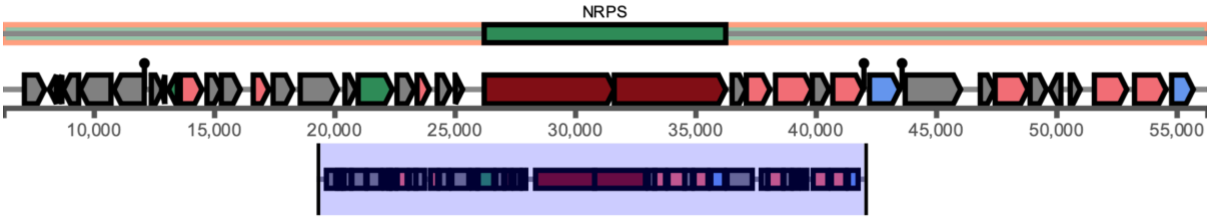

Figure S3. Location: 6,241 – 56,277 nt. (total: 50,037 nt); NRPS

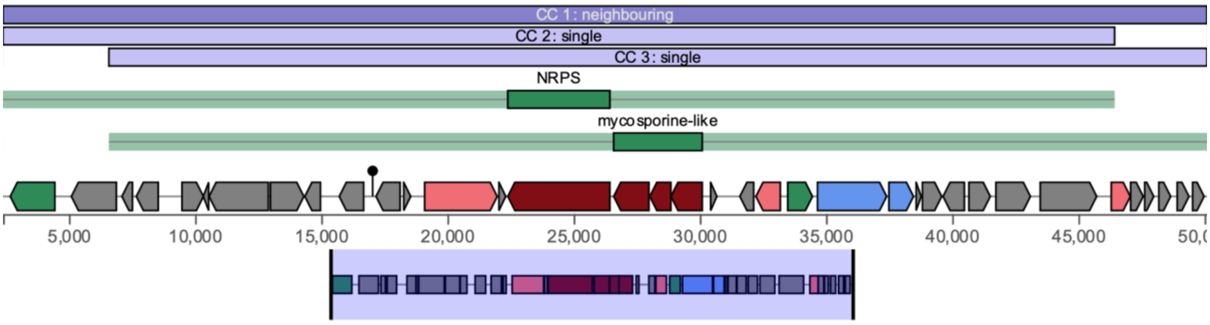

Figure S4. Location: ,392 – 50,097 nt. (total: 47,706 nt); NRPS, mycosporine-like

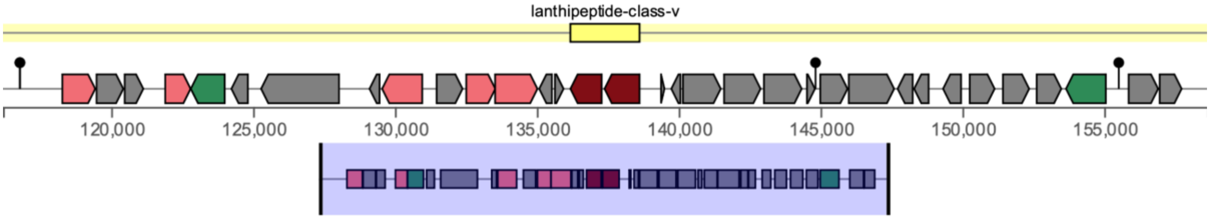

Figure S5. Location: 116,186 – 158,622 nt. (total: 42,437 nt.); lanthipeptide-class-v

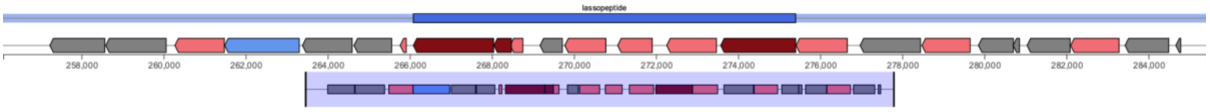

Figure S6. Location: 256,087 – 285,402 nt. (total: 29,316 nt.); lassopeptide

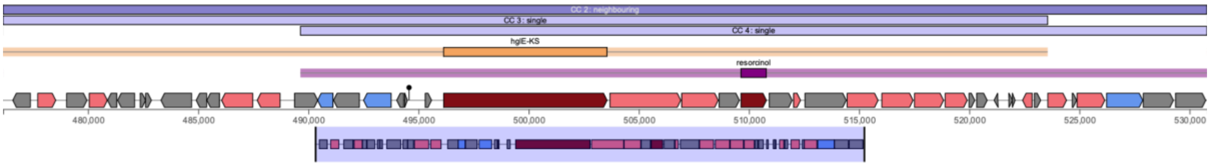

Figure S7. Location: 476,142 – 530,793 nt. (total: 54,652 nt.); hglE-KS, resorcinol

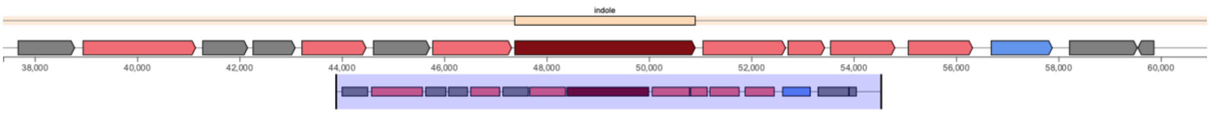

Figure S8. Location: 37,379 - 60,903 nt. (total: 23,525 nt); indole

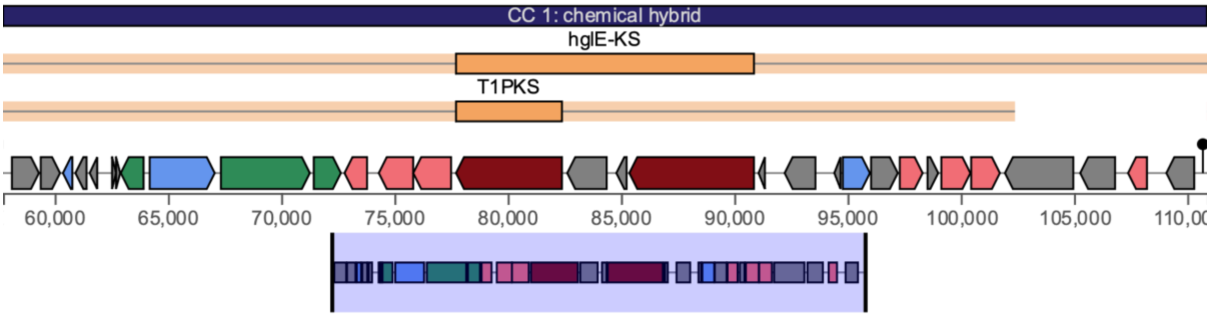

Figure S9. Location: 57,721 - 110,872 nt. (total: 53,152 nt); hglE-KS, T1PKS

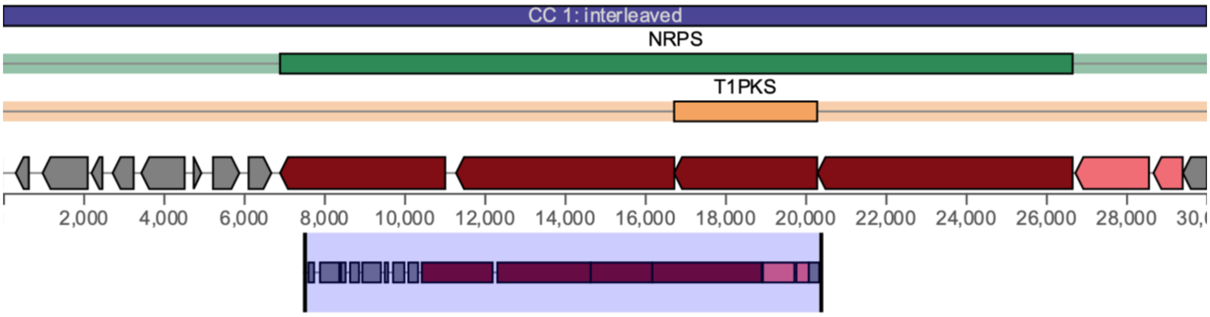

Figure S10. Location: 1 - 30,017 nt. (total: 30,017 nt); NRPS, T1PKS

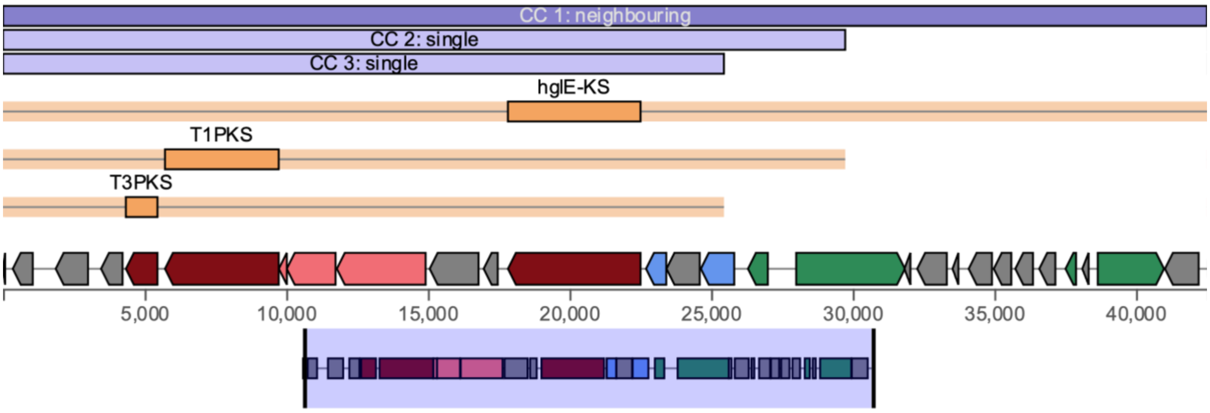

Figure S11. Location: 1 - 42,508 nt. (total: 42,508 nt); hglE-KS; T1PKS, T3PKS

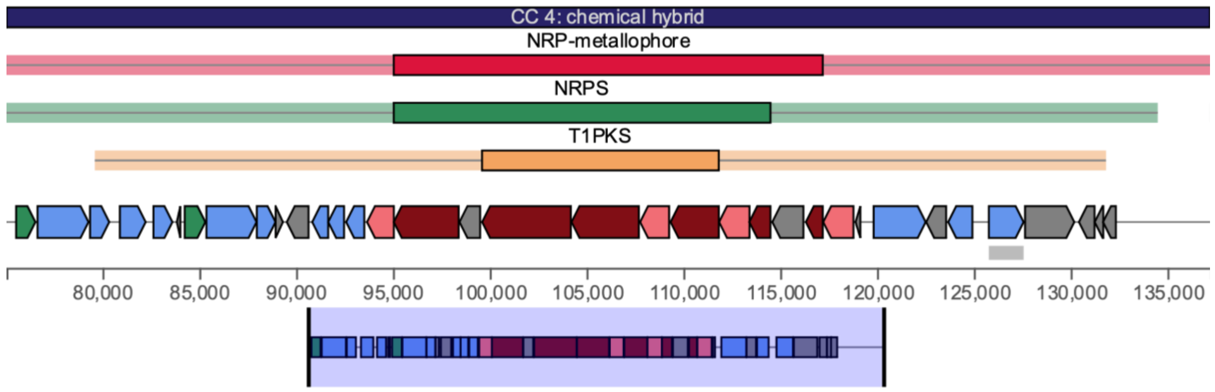

Figure S12. Location: 75,045 - 137,198 nt. (total: 62,154 nt); NRP-metallophore, NRPS, T1PKS

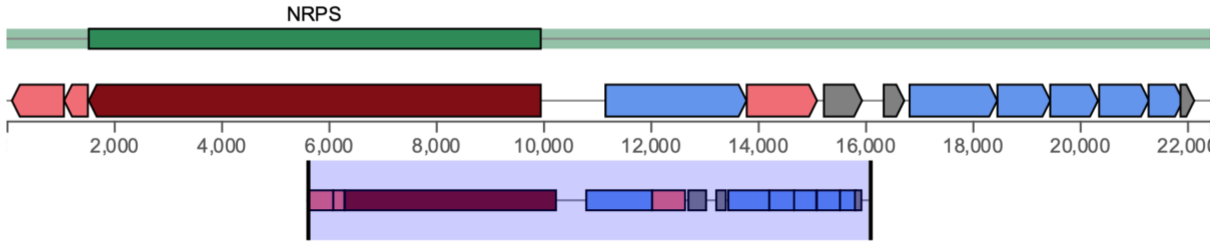

Figure S13. Location: 1 - 22,428 nt. (total: 22,428 nt); NRPS

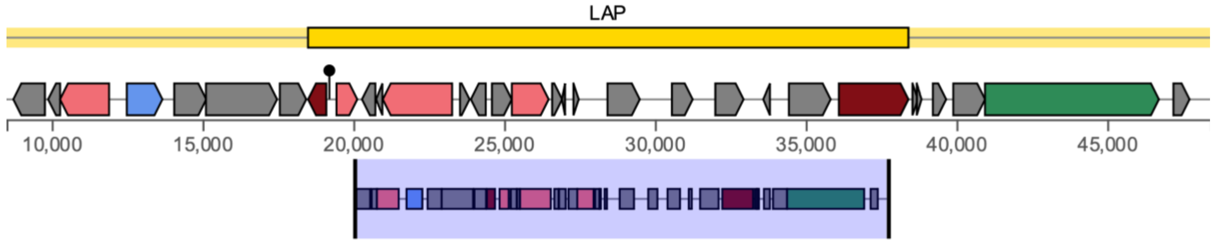

Figure S14. Location: 8,501 - 48,408 nt. (total: 39,908 nt); LAP

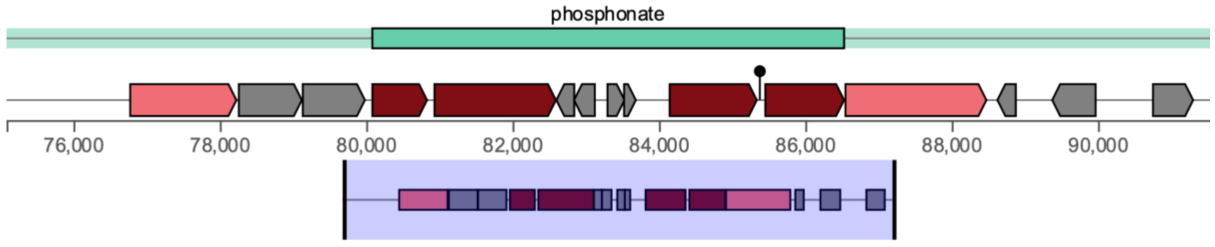

Figure S15. Location: 75,086 - 91,531 nt. (total: 16,446 nt); phosphonate

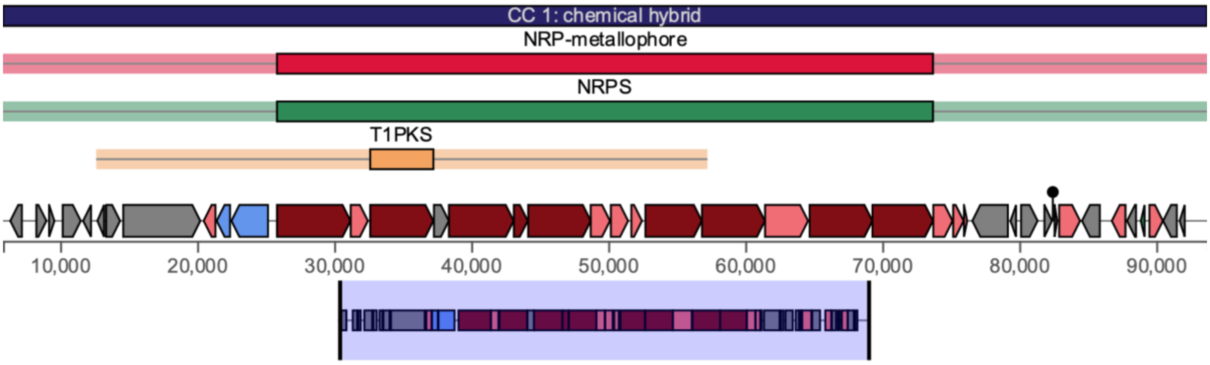

Figure S16. Location: 5,833 - 93,705 nt. (total: 87,873 nt); NRP-metallophore, NRPS, T1PKS

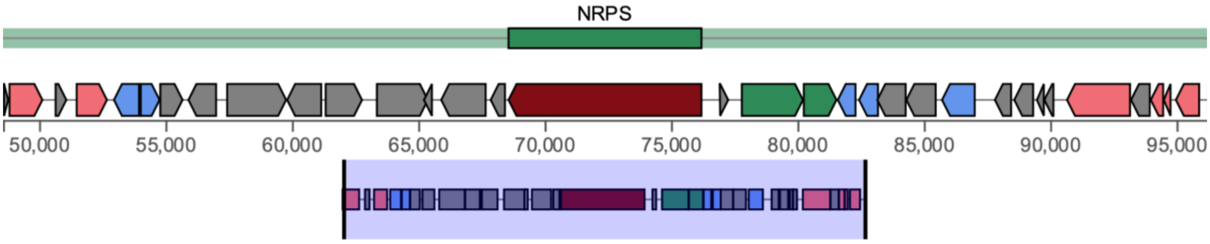

Figure S17. Location: 48,574 - 96,205 nt. (total: 47,632 nt); NRPS

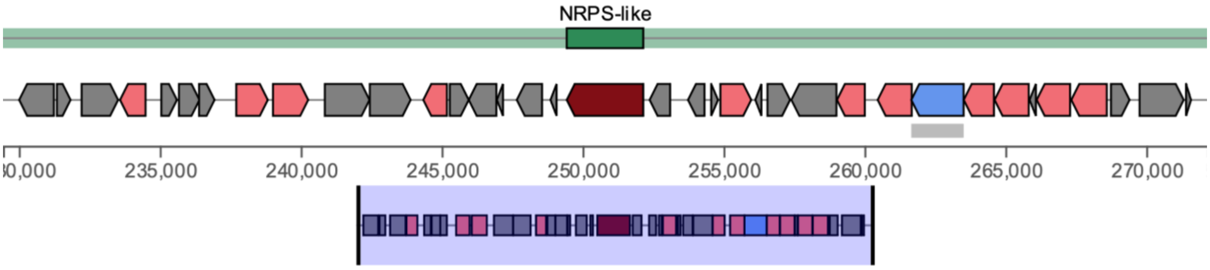

Figure S18. Location: 229,442 - 272,156 nt. (total: 42,715 nt); NRPS-like
